# Author Correction: Droplet superpropulsion in an energetically constrained insect

**DOI:** 10.1038/s41467-023-40159-3

**Published:** 2023-07-20

**Authors:** Elio J. Challita, Prateek Sehgal, Rodrigo Krugner, M. Saad Bhamla

**Affiliations:** 1 School of Chemical & Biomolecular Engineering, Georgia Institute of Technology, 311 Ferst Drive NW, Atlanta, GA 30332 USA; 2 George W. Woodruff School of Mechanical Engineering, Georgia Institute of Technology, 801 Ferst Drive NW, Atlanta, GA 30318 USA; 3 United States Department of Agriculture, Agricultural Research Service, San Joaquin Valley Agricultural Sciences Center, Parlier, CA 93648 USA

**Keywords:** Motility, Fluid dynamics, Biological physics

Correction to: *Nature Communications* 10.1038/s41467-023-36376-5, published online 28 February 2023

The original version of this Article contained an error in Equation (2) for fluidic pumping, and incorrectly read: ${\eta }_{{{{\rm{ex}}}}}=\frac{\rho {u}^{2}}{2}+\frac{32\mu lu}{{d}^{2}}+\frac{4\gamma }{d}$. The correct form of Equation (2) is: ${\eta }_{{{{\rm{ex}}}}}=\frac{32\mu lu}{{d}^{2}}+\frac{4\gamma }{d}$. This has been corrected in the PDF and HTML versions of the Article. In conditions of dominant viscous forces, characterized by a low Reynolds number (as is the case for sharpshooter insects), the inertial term $\frac{\rho {u}^{2}}{2}$ can be neglected since its contribution remains insignificant (<1%) in this low Reynolds regime. Importantly, this adjustment does not alter the quantitative estimates, figures presented, or conclusions of this Article.

The original version of the Supplementary Information associated with this Article contained an error in section “V. Energetics of excretion”, subsection “C. Hydrodynamics of excretion in glassy-winged sharpshooters”, under the heading “1. Model description and assumptions” which incorrectly read “We ignore potential viscous losses at 0 < Re < 1000 examined in constricted flows through very small orifices (<1 mm) [31–33]. The equation for the steady-state flow required to pump water across the hindgut of the insects may be written as follows [20]: ${P}_{{{{\rm{f}}}}}=\frac{\rho {u}^{2}}{2}+\frac{32\mu lu}{{d}^{2}}+\frac{4\gamma }{d}$.” The correct version states “We ignore potential inertial forces or viscous losses at 0< Re <1000 examined in constricted flows through very small orifices (<1 mm) [31–33]. The equation for the steady-state flow required to pump water across the hindgut of the insects may be written as follows [20]: ${P}_{{{{\rm{f}}}}}=\frac{32\mu lu}{{d}^{2}}+\frac{4\gamma }{d}$.” The HTML has been updated to include a corrected version of the Supplementary Information.

## Supplementary information

Supplementary information

