# Supplementary material for: Droplet superpropulsion in an energetically constrained insect
Source: Nat Commun. 2023 Feb 28;14:860. doi: 10.1038/s41467-023-36376-5 (PMC9975225; doi:10.1038/s41467-023-36376-5)
Supplement: Supplementary file 1 — Supplementary Information [file 41467_2023_36376_MOESM1_ESM.pdf]

Supplementary Information - Droplet superpropulsion in an energetically constrained insect

Elio J. Challita,[1,2] Prateek Sehgal,[1] Rodrigo Krugner,[3] and M. Saad Bhamla[1,*]

[1]*School of Chemical & Biomolecular Engineering, Georgia Institute of Technology, 311 Ferst Drive NW, Altanta, GA 30332, USA*

[2]*George W. Woodruff School of Mechanical Engineering, Georgia Institute of Technology, 801 Ferst Drive NW, Altanta, GA 30318, USA*

[3]*United States Department of Agriculture, Agricultural Research Service, San Joaquin Valley Agricultural Sciences Center, Parlier, CA 93648, USA*

(Dated: June 23, 2023)

Contents

I. **Anatomy of sharpshooter** 1
  A. Description of anal stylus 1
  B. Fluorescence, confocal imaging and μCT 2
  C. Potassium hydroxide (KOH) clearing 2

II. **Kinematics of droplet catapulting** 2
  A. Field Data at United States Department of Agriculture (USDA) 2
  B. Data analysis 2
  C. Hairless sharpshooters 3
    1. Experimental details 3
    2. Dynamics of droplet ejection 3

III. **Superpropulsion** 4
  A. Two-spring model: assumptions and simplifications 4

IV. **Droplet size Analysis** 4
  A. Surface properties of anal stylus 4
  B. Ejection dynamics of droplets on vibrating parahydrophobic surfaces 4

V. **Energetics of excretion** 5
  A. Ecology of excretion in organisms 5
  B. Excretion in xylem-feeders 5
  C. Hydrodynamics of excretion in glassy-winged sharpshooters 5
    1. Model description and assumptions 5
    2. Dripping and jetting 6

VI. **Table of other organisms & Scaling** 6

VII. **Supplementary figures** 6

Supplementary References 14

* **

I. Anatomy of sharpshooter

A. Description of anal stylus

Using optical microscopy, we take a close look into the anal tube located at the apex of the pygofer of a glassy-winged sharpshooter. This anal tube consists of the anal stylus, epiproct, and paraproct along with a large membranous clear structure. The anal stylus (anal ligulae) is a complex sclerotized appendage covered with mechanosensory hairs, coeloconic sensilla, and large aggregations of microscopic secretory granules called brochosomes [1]. The epiproct is a ringed sclerotized segment with two symmetrical projections embedded in the basal membranous structure with a sharp protrusion next to the anus [2]. The basal membranous structure bearing the epiproct can telescope, allowing multiple degrees of freedom for the insects to move around their stylus during the different excretion stages and possibly aim the trajectory of the ejected droplet. A narrow canal spans along the longitudinal axis of the anal stylus, which carries the pumped liquid waste after exiting the anus. Owing to its closed-ended groove-like geometry, it directs the pumped fluid in the direction perpendicular to the stylus, eventually forming a droplet excreta before its eventual ejection. We use fluorescence and confocal microscopy to identify the involved structures' material composition. We identify the existence of a blob of the rubbery protein resilin sandwiched between the base of the stylus and the sclerotized paraproct. Resilin is a rubbery protein structure employed by leafhoppers and other insects to mechanically store energy and quickly release it during various explosive movements [3]. The salient existence of the resilin structure at the base of the stylus suggests that the flicking movement is a spring-assisted mechanism. We corroborate this by manually rotating and releasing the anal stylus into the ventral direction using a pulled micropipette (Supplementary Video I). Once released, the anal stylus snaps back into its original position, suggesting that this movement is mediated by the elastic resilin, not an adductor muscle.

B. Fluorescence, confocal imaging and μCT

The anal stylus of dried Glassy-winged sharpshooter insects *Homalodisca vitripennis* was imaged using Zeiss LSM 780/Elyra PS.1 system. Autofluorescence of resilin was reported at excitation 405 nm laser line and bandpass emission 400 - 420 nm [4]. We further identify the autofluorescence of resilin using the DAPI filter-set with fluorescence microscopy (Nikon eclipse Ti2). The full protocol is discussed in detail in Michels & Gorb [4].

C. Potassium hydroxide (KOH) clearing

To further understand the anatomy of the stylus and anal tube of sharpshooters, we expose the exoskeleton by dissolving the soft and membranous tissues, such as the resilin of the sharpshooter specimen, with a potassium hydroxide (KOH) solution. The abdomen and hindgut of a glassy-winged sharpshooter insect are isolated and soaked in 10% aqueous KOH solution for 12 hours to clear the soft membranous tissues [5]. As a result, soft membranous tissues such as resilin are dissolved, and the insect's exoskeleton is exposed. Manually bending the cleared stylus uncovers a pivot point at the base of the stylus. However, the rotating restoring force was significantly less pronounced when compared to the untreated specimen, and the stylus did not completely bend back to its original location.

II. Kinematics of droplet catapulting

A. Field Data at United States Department of Agriculture (USDA)

Like other leafhoppers, sharpshooter insects are known for their maneuverability on stems and leaves and ability to successfully conceal themselves while they secretly feed and 'shoot' their liquid droplet excreta (Hence their name 'sharpshooter'), posing challenges to data collection. Therefore, high-speed imagery of the excretion of two sharpshooter species (Glassy-winged sharpshooters, *Hemalodisca vitripennis* and Blue-green sharpshooter, *Graphocephala atropunctata*) were mainly collected at the USDA-Agricultural Research Service (ARS) in Parlier, California. Other data, mainly from red-banded sharpshooter, *Graphocephala coccinea* were collected from a backyard in Atlanta, Georgia, while they were feeding from a basil plant. A Chronos 1.4 high-speed camera connected to a Canon MPE 65 mm Macro lens was used for high-speed video recording with a field portable Zaila high-intensity light. Data were collected by ensuring that both the droplet and the stylus stayed in focus with the camera during ejection. Both systems' resulting trajectory and displacement are assumed to be in 2D.

B. Data analysis

Data analysis is performed using MATLAB and ImageJ (FIJI). We manually track the angular displacement of the stylus $\theta_s(t)$ with respect to the axis running along the insect's body. The stationary origin is chosen at the intersection of the stylus and body axes. Note that in some instances, the basal area of the stylus is not completely stationary during the movement. To offset the kinematics of this relative movement, we separately track the base of the stylus with respect to a stationary point along the insect's body and subtract it from the displacement of the stylus. Raw data are filtered using a moving average with a window equal to 3 and 5. The angular velocity $\dot{\theta} = \partial_t \theta$ and angular acceleration $\ddot{\theta} = \partial_t \dot{\theta}$ are calculated and shown in Fig. 1 of the main text. The maximum linear speed of the stylus is calculated as $V_s = L_s \dot{\theta}_{max}$. Similarly, we manually track the centroid of the droplet after take-off. Ejection is defined as when the droplet becomes completely detached from the stylus after droplet extension. We measure the 2D location of the droplet $P(x_e, y_e)$ at take-off time t_e and at $(t_e - dt)$, $(t_e + dt)$. For each of these frames, we skip three frames $(3 \times dt)$ and measure the centroid's final location $P(x_f, y_f)$. The displacement is calculated by measuring the Euclidean distance $d = \sqrt{(x_f - x_e)^2 + (y_f - y_e)^2}$ of the droplet. The ejection velocity is obtained by averaging the three velocities as $V_d = \partial_t\ d$. Both the kinematic values of the stylus and droplet are verified with DeepLabCut [6], which showed similar trends and velocity ratios V_d/V_s.
To extract the temporal dynamics of the stylus, we examine the shape of the angular displacement curve. We approximate the displacement of the stylus as a step function and the data are fitted using the MATLAB curve fitting tool to the following kinematics models $\theta(t) = a \times \text{erf}(b \times t + c) + d$ where $a,\ b,\ c$ and d are fitting factors. This model circumvents the challenge of estimating the beginning and end of the movement. To estimate the effective frequency of the stylus movement, a sinusoidal function $\theta(t) = \theta_o \sin(2\pi f_s t + \phi)$ may be fitted to the angular speed curve of the raw data or the second derivative of the step function (in the case of lower fps < 3000 or noisy data). Another way to calculate the frequency f_s is to directly extract it from the kinematics data by considering the peak-to-peak duration in the angular acceleration curve, which corresponds to half the period of the movement. However, this method is more susceptible to the higher level of noise that naturally results from taking the second derivative of displacement and is mainly used in hairless kinematics, where the angular speed curve resembles a sinusoidal function. In any of these cases, the variation captured within the calculated frequencies f_s does not change the conclusions reached in this work. The frequency of droplets is estimated as the second mode of the Rayleigh frequency of freely vibrating droplets $f_o = (1/2\pi)\sqrt{8\gamma/\rho R_o^3}$. The diameter D_o $(= 2R_o)$ is estimated from high-speed videos

Parameter		Description	GWSS	GWSS hairless	BGSS	RBSS
**Droplet**						
	m_d	Mass	$26 \times 10^{-8}\ kg$	$7.6 \times 10^{-8}\ kg$	$5.56 \times 10^{-8}\ kg$	$2.6 \times 10^{-8}\ kg$
	D	Diameter	$725 \pm 188\ \mu m$	$523 \pm 48\ \mu m$	$470 \pm 45\ \mu m$	$369 \pm 19\ \mu m$
	$V_{d,e}$	Ejection velocity	$0.32 \pm 0.1\ m/s$	$0.45 \pm 0.07\ m/s$	$0.35 \pm 0.09\ m/s$	$0.74 \pm 0.05\ m/s$
	$\dot{\theta}_d$	Angular velocity	$\sim 175\ rad/s$	$\sim 2.4 \times 10^3\ rad/s$		
	E_K	Translational kinetic energy	$9.6 \pm 2.6 \times 10^{-9}\ J$	$7.34 \pm 1.8 \times 10^{-9}\ J$	$1.67 \pm 0.5 \times 10^{-9}\ J$	$7 \pm 10^-9\ J$
	E_R	Rotational kinetic energy	$\sim 6.6 \times 10^{-11}\ J$	$\sim 2.3 \times 10^{-9}\ J$		
**Stylus**						
	L_s	Average stylus length	$428\ \mu m$	$428\ \mu m$	$266\ \mu m$	$340\ \mu m$
	$V_{s,max}$	Maximum velocity	$0.23 \pm 0.07\ m/s$	0.89 ± 0.2	$0.25 \pm 0.07\ m/s$	$0.63 \pm 0.05\ m/s$
	$A_{s,max}$	Maximum Acceleration	$\sim 14 - 28\ g$		$\sim 38 - 64\ g$	

TABLE I: Summary of kinematic properties of GWSS, GWSS hairless, BGSS, RBSS. Maximum values are calculated for the tip of the stylus

by taking the average of the vertical and horizontal diameters, while $\gamma = 72 \times 10^{-3}\ N/m$ is the surface tension of water at $25°C$ and $\rho = 996\ kg/m^3$ is the density of water.

C. Hairless sharpshooters

One salient feature of the anal stylus is the spread of large mechanosensitive sensilla with tiny coeloconic sensilla (hair) along its surface (Main text Fig. 3a). These hair structures help sharpshooters select host plants and detect the moisture content in plants by acting as hygrosensors [7]. To understand their role in excretion, we perform ablation experiments where we carefully trim the tip of the hair structures located at the apex of the anal stylus of glassy-winged sharpshooters using micro scissors. After trimming the sensilla, insects were maintained on host plants for a 48-h acclimatization period before being used in recordings. We observe that 'hairless' sharpshooters eject smaller droplets having diameters of $D_o^- = 523 \pm 48\ \mu m$ at higher speeds for both their stylus and droplet with $V_s^- = 0.89 \pm 0.2\ m/s$ ($n = 4$, $N = 10$) and $V_d^- = 0.45 \pm 0.07\ m/s$ respectively (Supplementary Figure 4). The calculated speed ratio is $\lambda^- < 1$ indicating that droplet ejection in hairless sharpshooters does not fall in the superpropulsion regime.

1. *Experimental details*

Glassy-winged sharpshooter insects were captured and sedated under CO2. The hairs at the tip of the stylus were carefully snipped using scissors while avoiding damage to the stylus or the overall insect. The 'hairless sharpshooters' were released in an isolated and confined area.

2. *Dynamics of droplet ejection*

In hairless sharpshooters, video analysis shows that droplet extension and take-off occur after the stylus stops moving (Fig. 3c). However, droplet ejection occurs in control sharpshooters while the stylus is still moving.
In addition, we observe significant droplet deformation in hairless sharpshooters. Deformation is quantified as the droplet height $h(t)$ normalized with the initial height $h_o \approx D_o$. At ejection, $h/h_o|_e$ is ~ 1.6 which is 30% more than droplets ejected in control sharpshooters (Supplementary Information, Section 3).
Moreover, the ejected droplets undergo a substantial rate of rotation around their axis with an angular velocity $\Omega^- \sim 2.4 \times 10^3\ rad/s$ compared to $\Omega^+ \sim 170\ rad/s$ (Supplementary Video I).
To gauge the relative impact of rotation, we consider both the translational energy E_t and rotational energy E_r transferred to the droplet from the rotating stylus. In hairless sharpshooters, the energy ratio of ejected droplets $(E_r/E_t)^- = (r_g\Omega/V_d)^2$ is ~ 0.2 (where $r_g = R_o/\sqrt{10}$ is the radius of gyration of the spherical droplet). In control sharpshooters, however, energy is almost entirely transferred to translational energy with $(E_r/E_t)^+ \sim 10^{-2}$. This substantial increase in energy lost to droplet rotation implies that the rotational aspect of the stylus (previously ignored) plays an important role in dictating droplet dynamics during ejection. For instance, the increase in the frequency of the stylus f_s may lead to a considerable increase in the effect of inertial forces such as the Euler and centrifugal force ($\propto f_s^2$) in the rotating frame of reference of the stylus. Extreme cases of these inertial forces may lead to movement of the droplet (slip) in the parallel direction to the stylus, only to be opposed by capillary adhesion. As a result, the moving stylus may cause a net tangential force causing the droplet to rotate, similar to how an off-centered strike to a billiard ball would make it sidespin. Furthermore, given the significant size of the hairs (at least 40% the length of the stylus), cutting the hair structures shortens the length of the stylus, possibly exacerbating these inertial and geometrical effects.

III. Superpropulsion

A. Two-spring model: assumptions and simplifications

The two-spring oscillator model is chosen for its simplicity in describing the droplet's vibrations and resilin's dynamics. It is not meant to give a one-to-one representation of the system but to reveal critical features of two coupled oscillating systems, such as temporal matching and ejection speed.
The complex dynamics of bouncing droplets have been previously modeled using different spring-based models, such as the 'bouncing model' whereby a droplet is estimated as a Kelvin-Voigt material [8]. A molecular dynamics approach is taken to evaluate the contact mechanics between the actuator and the droplet. The contact force is modeled as a damped spring only active during the compression of the upper spring and null otherwise. Similar to the two spring models, the bouncing model predicts superpropulsion within a different frequency range and λ vs. f_o/f profiles (Supplementary Figure 7).
Another simplification in this model is that we ignore any potential ejection dynamics arising due to the sessile nature of the formed water droplet. Sharp *et al.* showed that the resonant frequency response of sessile droplets depends on the contact angle such $f_i = \frac{\pi}{2}\sqrt{(i^3\gamma\cos^3\theta_e - 3\cos\theta_e + 2)/(24 m_d \theta_e^3)}$ where i represent the ith vibrational mode of the droplet, and θ_e is the contact angle between and m_d is the mass of the quasi-spherical droplet [9]. This equation has a similar functional form to the classical equation developed by Lord Rayleigh for the natural frequency of free oscillating droplets (which is used in this work for $n = 2$), $f_n = \frac{1}{2\pi}\sqrt{n(n-1)(n+2)\gamma/3\pi m_d}$ where $n = 2, 3, 4...$ corresponds to the mode number [9]. Adjusting the natural frequencies f_o by considering the contact angle θ_e would shift the theoretical, computational, and field data, but the same trends are still conserved.

IV. Droplet size Analysis

A. Surface properties of anal stylus

Another important aspect of the anal stylus is its observed complex hydrophilic and hydrophobic surface properties. In addition to its role in droplet excretion, the anal stylus secretes protein-lipid buckyball-like particles ($0.2 - 0.6\ \mu m$) called brochosomes. These microscopic structures are used by leafhoppers to actively form a protective layer covering their integuments and eggs after oviposition [10]. Furthermore, brochosomes coatings render surfaces superhydrophobic [11]. As a result, fully formed sessile droplets on the anal stylus possess an apparent high contact angle $\theta_a \sim 140^\circ - 150^\circ$ as measured from side-view image analysis (Supplementary Figure 7). However, excreted droplets do not roll down or slip due to gravity once created, regardless of the orientation of the stylus or during abdomen shaking during mating calls.

B. Ejection dynamics of droplets on vibrating parahydrophobic surfaces

A patterned hydrophobic/hydrophilic surface yields interesting properties related to the droplet shape during fluidic pumping and ejection. As the fluid volume increase during pumping, the excreted fluid transitions from being a thin fluidic film to a sessile quasi-spherical droplet while having a constant contact surface S between the fluid and the stylus (determined by the surface area S) without wetting and engulfing the stylus (due to resilin, hair, and brochosomes) (Supplementary Figure 7). Meanwhile, the equilibrium (young's) contact angle θ_e ($\approx \theta_a$) increases as $\theta_e \approx \sin^{-1}(1 - 3\pi h^3/(\pi h^3 + 3V\!\!\!/_c)$ where $V\!\!\!/_c = \frac{1}{6}\pi h(3L_s^2 + h)$ and L_s is the length of the stylus for a spherical cap approximation ($Bo \sim 10^{-1}$). As the droplet grows with a droplet height $h(t)$ approaching $D_o \approx 725\ \mu m$ (GWSS), θ_e increases to 150° and the work required to overcome surface adhesion $E_s \propto S\gamma(1 + cos\theta_e)$ decreases (Supplementary Figure 8). To overcome surface adhesion, the stylus imparts kinetic energy to the droplet while scales as $E_k \propto \frac{1}{2}\rho V\!\!\!/(2\pi f A)^2$ where $A = \frac{1}{2}L_s\Delta\theta$ is the half peak to peak vibrational amplitude set by the arclength of the traveling stylus[12]. Taking the ratio E_k/E_s yields a critical frequency ratio $(f_o/f)_c$ required to detach a droplet $(f_o/f)_c \sim 4A\sqrt{\pi/(3S(1 + \cos\theta_a))} \leq 5.2 - 13.12$ for a $\theta_a = 100^\circ - 150^\circ$ respectively.

Given their inherent lower f_o and E_s and constant contact surface S, these results suggest large sessile droplets formed on parahydrophobic surfaces are easier to remove through kinetic energy ($\propto f$). In addition, for a given actuator velocity V, surface tension-dominated larger droplets ($Bo < 1$ & $We < 1$) are easier to deform as $dh \sim R^{3/2}V$ [13] allowing them to store potential energy through surface deformation and to take-off at lower critical velocities V_c and accelerations Γ_c [14, 15].

Alternatively, CFD simulations of the ejection dynamics of droplets with various contact angles reveal that droplet detachment occurs when the contact line L between the droplet and the substrate approaches zero while the relative speed between the droplet and the substrate is greater than or equal to zero (Supplementary Figure 9). An in-depth analysis of superpropulsion and droplet ejection would consider the movement of the contact line and shape analysis of the droplet during ejection.

V. Energetics of excretion

A. Ecology of excretion in organisms

Food consumption and waste elimination are two essential functions of biological organisms. While these biological activities come hand-in-hand, more attention had been given to foraging and feeding. Despite having profound physiological, ecological and evolutionary implications, the science of biological waste management across taxa remains comparatively underexplored [16]. Insects, in particular, exhibit a wide range of ingenious waste disposal strategies determined by their environment, diet, and generalized lifestyle. In addition to the natural need to maintain good hygiene, some insects repurpose their waste products to achieve other functionalities essential to their survival and proliferation. For instance, termites use their excrement as building blocks for their nests [17]; Larvae of tortoise beetles Chrysomelidae, Cassidinae) construct fecal shields on their back to deter potential predators such as ants [18]; Old borers exploit volatile chemicals in their frass to attract mates increasing their chances of reproduction [19].

Why do sharpshooters catapult droplet excreta? Large animals urinate by generating jets or sheets of urine, except for small animals such as rodents and bats, which produce a series of small breakup droplets due to their small bladder [20]. The ballistic ejection of excrement is not uncommon among insects. Many insect species may be described as 'frass-shooters', 'butt-flickers', and 'turd-hurlers' that innovated unusual strategies to launch away both liquid and solid excrements [16]. For instance, frass-shooting skipper larvae employ biological latches on their anal plates coupled with a hydrostatic blood pressure buildup to propel solid pellets up to 38 times their body length with a speed larger than 1.5 m/s. Some species of noctuids violently shake their abdomen as they release their frass pellets, and some geometrid larvae use their thoracic legs to kick away their frass pellets [16]. The need to create a large distance between insects and their waste is mostly observed in shelter-dwelling or site-faithful insects, which are typically pressured to maintain hygiene at the site/shelter to avoid the growth of pathogens and reduce chemical cues for potential predators.

B. Excretion in xylem-feeders

Xylem-feeding insects have additional sets of energetic constraints mainly imposed by the nature of their liquid diet. Xylem sap is inherently extremely low in nutritional compounds and is under negative tension, making it exceedingly challenging to extract. To extract significant nutrients, xylem-suckers typically consume large amounts of sap and excrete large volumes of liquid waste, reaching up to 300 times their body weight in 24 hours [21, 22]. However, pumping fluids (both internally and externally) requires energy. Specifically, feeding on xylem sap is especially energetically taxing. Sharpshooter insects have developed unique digestive structures called filter chambers capable of concentrating and separating nutrients with extremely high efficiency to maximize nutrient extraction. The resulting liquid waste is 99% water [23]. Novotny and Wilson argue that xylem feeders have an evolutionary minimum in body size. They showed that it is energetically taxing for smaller insects to create a suction pressure gradient while feeding given their isometrically narrower feeding apparatus and high metabolic rate [24].

C. Hydrodynamics of excretion in glassy-winged sharpshooters

1. *Model description and assumptions*

The hydrodynamics of excretion in glassy-winged sharpshooter (GWSS) insects is investigated.
We model the excretion of fluidic waste through the anal tube as a pressure-driven flow of liquid water (Density $\rho = 996\ kg/m^3$, Surface tension $\gamma = 72\ mN/m$ and Viscosity $\mu = 1\ mPa.s$) through a straight and circular cylinder having a cross-sectional diameter d and length l . Given little to no corrugations in the digestive tract leafhoppers as suggested by histological analysis [25, 26], the inner surface cylinder is considered smooth. Using micro-CT scans and high-speed videos, the dimensions of the cylinder are estimated, and the ratio $l/d \sim 0.12$ is inferred through micro-CT scans (Supplementary Figure 1) and assumed to be conserved across individual glassy-winged sharpshooters given isometric body variations in leafhoppers [24].

The volumetric flow rate Q is calculated as the ratio of the final volume of the droplet over its formation duration $Q = \forall/\Delta t = 0.3 \pm 0.22\ \mu L$. The speed of the exiting flow $u = Q/A = 0.39 \pm 0.08\ m/s$ where A is the average circular cross-sectional area of the stylus and the rectum estimated as $A = \pi d^2/4$. The speed of the excretion falls within the same velocity ranges reported for the suction xylem sap $(0.02 - 5\ m/s)$ [27, 28] . The flow is considered to be laminar ($Re = \rho u d/\mu\ \sim 10 - 60$) and at steady-state as the rate of growth of the droplet ($\sim h(t)^3$) is relatively constant (Supplementary Figure 6). Given the low Re, we ignore inertial effects due to the curvature of the bent anal stylus during droplet formation.

We estimate the effective diameter of the anal tube by fitting the cross-sectional area of the bottom of the stylus to a circle of diameter d and perimeter P. The canal's shape is irregular, with a more elliptical shape than a perfect circle. We find that the eccentricity $\epsilon = \sqrt{1 - a^2/b^2}$ along the length of the stylus and the rectum with an average hydraulic diameter $d = 4A/P$ where A is the cross-sectional area obtained from microCT (Main text Fig. 4a).

The pressure required to pump the liquid waste depends on the geometry of the excretion apparatus and the fluid properties. The pressure P_f required for fluidic pumping is induced by the lateral compression of the thin circular muscles in the hindgut [29]. This active pumping needs to overcome pressure loss due to inertia, viscous effects within the urethral tube, and capillary outside the tiny orifice [20]. The pressure drop due to viscous losses in a cylinder may be estimated from the Darcy-Weisbach equation for a laminar flow in a long cylindrical pipe as $P_{viscous} = 32\mu lu/d^2$. Surface tension forces at the orifice ($Bo = \rho g D^2/\gamma \sim 10^{-1}$) generate a capillary pressure $P_{\text{capillary}} = 4\gamma/D$ [30]. Due to the minute size of the insects, we ignore changes in hydrostatic pressure during droplet formation. We ignore potential inertial forces or viscous losses at $0 < Re < 1000$ examined in constricted flows through very small orifices ($< 1\ mm$)[31–33]. The equation for the steady-state flow required to pump water across the hindgut of the insects may be written as follows [20]: $P_f = 32\mu lu/d^2 + 4\gamma/d$
We calculate the average pressure to form a droplet for a sharpshooter insect (*Homalodisca vitripennis*) $P_f \approx 14\ kPa$. Interestingly, this is almost three times the bladder pressure excreted by mammals and small animals during urination [20] but below pressures of $\sim 60\ kPa$ exerted within the respiratory system of animals ranging from small insects such as mosquitoes to large mammals such as elephants [34].

Maintaining a net positive energy gain from feeding on xylem fluid is critical for the insects' functioning and survival [27, 35]. Andersen *et al.* (1992) estimated that glassy-winged sharpshooters (formerly *Homalodisca coagulata*) generates a net positive energy per unit fluid volume (η_{in}) to range from 0.2 to 8.2 J/cm^3 [27] (See discussion main text). Interestingly, feeding and excretion are observed to stop above a threshold xylem tension pressure of $\sim 2.1\ MPa$, possibly due to the inability of the cibarial pump to generate a large enough internal pressure that is also energetically justifiable. [36].

2. *Dripping and jetting*

The shape of water flowing out of a circular orifice (nozzle) of diameter d at speed u depends on the interplay of surface tension and inertial forces characterized by the Weber number $We = \rho u^2 d/\gamma$. At $We < 1$, the exiting fluid slowly forms a large pendant droplet with a diameter $D > d$. 'Dripping' occurs when the growing droplet's weight overcomes the nozzle's surface adhesion. In this case, the pendant droplet detaches and falls, leaving a residual portion of the fluid attached to the outer surface of the nozzle (e.g., a leaking faucet). At $We > 1$, the exiting fluid transitions into a jet where inertial forces overcome surface tension forces. As the speed of the fluid increase at $We > 8$, a continuous liquid jet forms at a critical Weber number $We > 8$ [37]. For simplicity, we consider the transition into a jetting regime at a critical Weber number $We = 1$ [38].

Why do sharpshooters not exploit gravity-induced dripping? First, 'dripping' forms droplets at low production rates. This mechanism is not ideal for sharpshooters since they need to pump out a large volume of fluids to extract sufficient nutrients. Second, due to their high feeding rate, sharpshooters are site-faithful and spend extended periods feeding in one location. Therefore, dripping might not be an ideal strategy for maintaining hygiene and preventing fouling. Third, dripping leaves a fluidic residue at the nozzle level, making it a poor strategy to expel large volumes of fluids and increase the likelihood of fouling[37]. Fourth, flinging droplet to large distances have been hypothesized to be an approach to avoid predator such as the tiny parasitic wasp *Cosmocomoidea* that may be attracted to chemical cues in the accumulation of their excreta. Fifth, field observations show that sharpshooter insects primarily feed with their heads facing the ground. Dripping droplets excreta might jeopardize the survival of the insects as the falling droplets may block the abdominal spiracles resulting in reduced inhaled air, potentially leading to asphyxiation. However, sharpshooter insects have been observed to feed under rainy conditions even when fully coated with rain water (Personal observation).

VI. Table of other organisms & Scaling

VII. Supplementary figures

Fig. S1 - Imaging and rendering of glassy-winged sharpshooter

Fig. S2 - Kinematic analysis of stylus during droplet ejection

Fig. S3 - Elasticity of the stylus

Fig. S4 - Droplet size and dynamics in hairless GWSS

Fig. S5 - Effect of damping on ejection kinematics in two spring model

Fig. S6 - Droplet growth on parahydrophobic and patterned stylus

Fig. S7 - Comparison between different droplet models for superpropulsion

Fig. S8 - Energy transfer and coupling limits predicted by the two spring model

Fig. S9 - Dynamics of droplet ejection on a vertically vibrating substrate having different contact angles

Organism	Orifice Diameter (m)	Speed (m/s)	Mechanism
**Cicada**	3.6e-4	0.9	Jetting
Ascobolus immersus [39]	3e-5	14	Jetting
Podospora anserina [39]	1.6e-4	21	Jetting
Pilobolus kleinii [39]	7.7e-4	9	Jetting
Basidiobolus ranarum [39]	8.69e-6	4	Jetting
Itersonilia perplexans[40]	8e-6	1.2	Droplet, Coalescence
Auricularia [40]	2.25e-6	0.8	Droplet, Coalescence
Sporobolomyces [40]	3e-6	2.3	Droplet, Coalescence
**Archerfish** [41]	2e-3	2	Jetting
**Bumblebee**	7.4e-4	0.35	Jetting
**Butterfly (Common Jay)**	6.5e-4	0.722	Jetting
**Human** [20]	6e-3	1	Jetting

TABLE II: Summary of parameters for organisms. Cicada data are based on our unpublished measurements made in the field. Bumblebee (https://www.youtube.com/watch?v=BeEoWReue4Y), Butterfly (https://www.youtube.com/watch?v=tlqXKcrueVA)

Fig. S10 - Matching between the CFD simulation and droplet propulsion from Raufaste *et al.*, PRL 2018

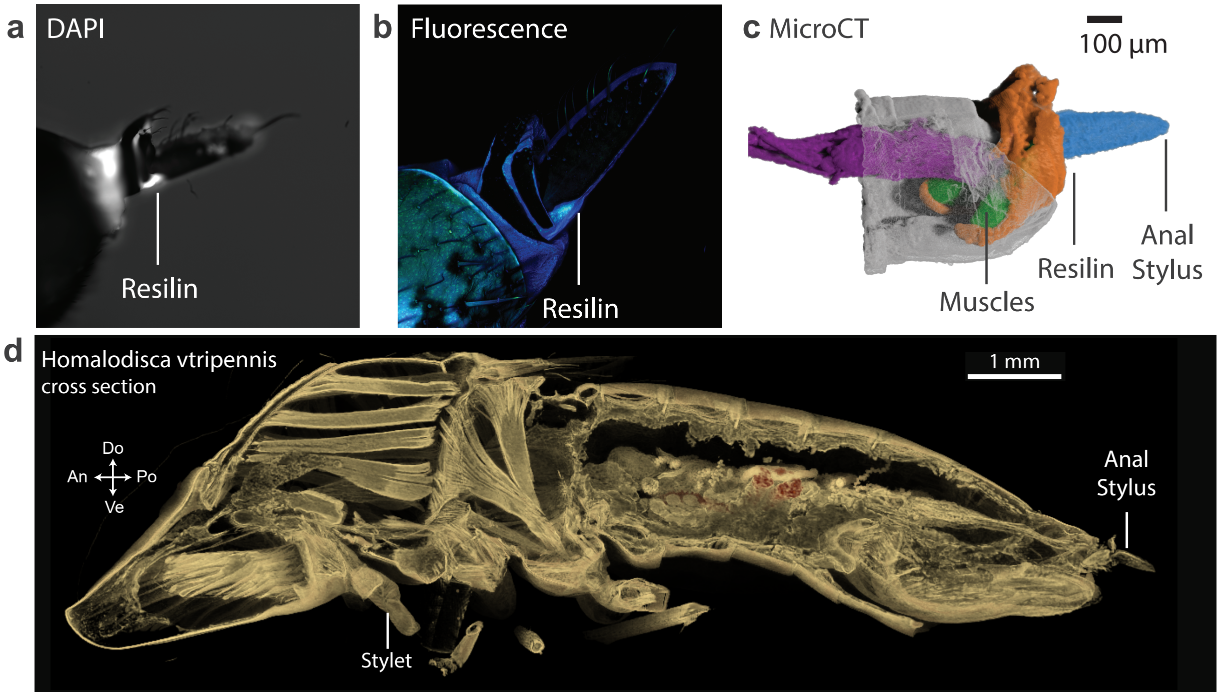

Fig. S1: Imaging and rendering of glassy-winged sharpshooter a. Fluorescence with DAPI filter of the sharpshooter anal stylus highlighting the resilin blob at the bottom of the stylus **b** Confocal image of the anal stylus **c** Close microCT render of the anal tube and anal stylus **d** Cross-sectional microCT of a glassy-winged sharpshooter *Homalodisca vitripennis* showing various parts of its digestive system

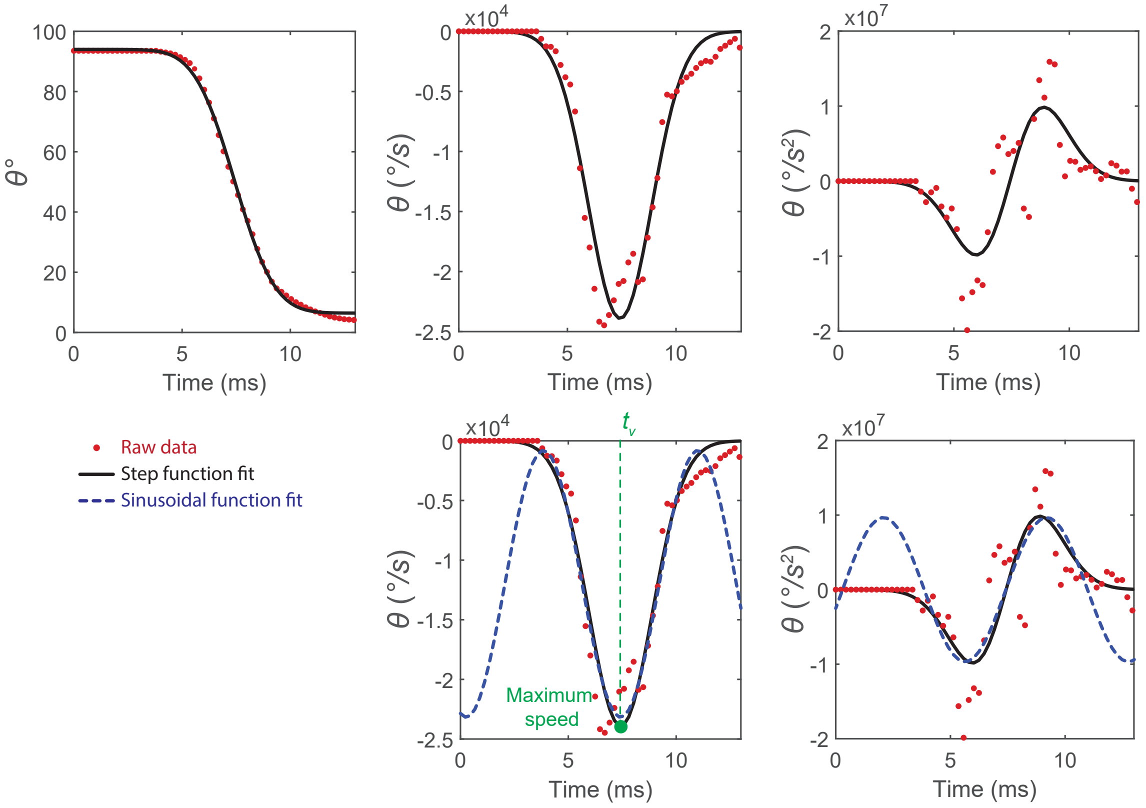

Fig. S2: Kinematic analysis of stylus during droplet ejection The angle θ between the stylus and the axis running along the body of the sharpshooter is tracked over time and smoothed with a moving average of size ranging between 3 and 5. A step function is fitted to the kinematic profile and used to extract the maximum velocity timestamp t_v and frequency of the stylus f. The frequency f is obtained by fitting a sinusoidal function to the angular velocity profile. See more details in Section II,B.

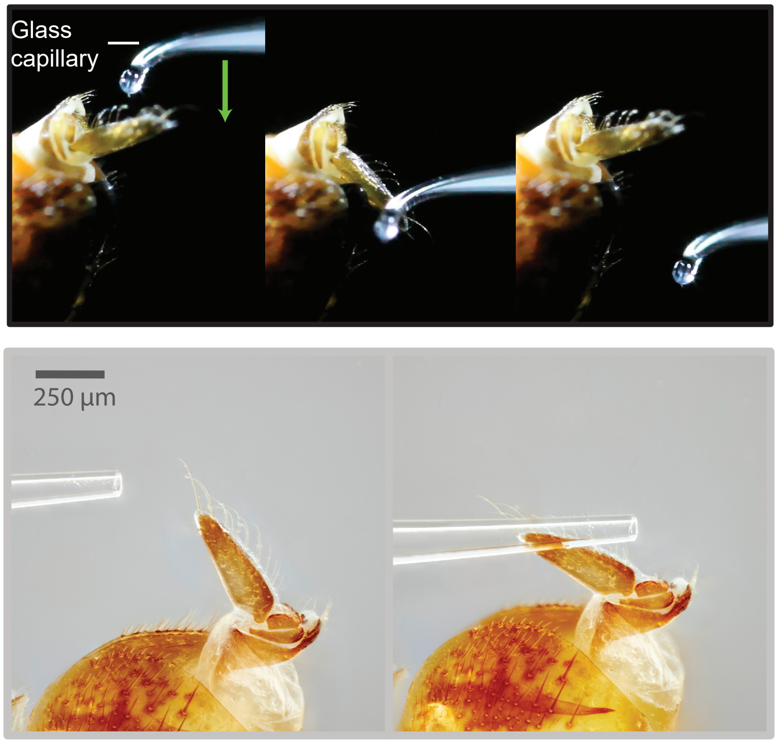

Fig. S3: Elasticity of the stylus is mediated by resilin a. We manually bend the stylus using a pulled capillary tube. The stylus returns to its initial position due to the elasticity of the resilin blob located at its bottom **b.** The elasticity of the stylus may be disrupted by dissolving soft tissues such as resilin with KOH. The anal stylus loses its elasticity and does not return to its initial position.

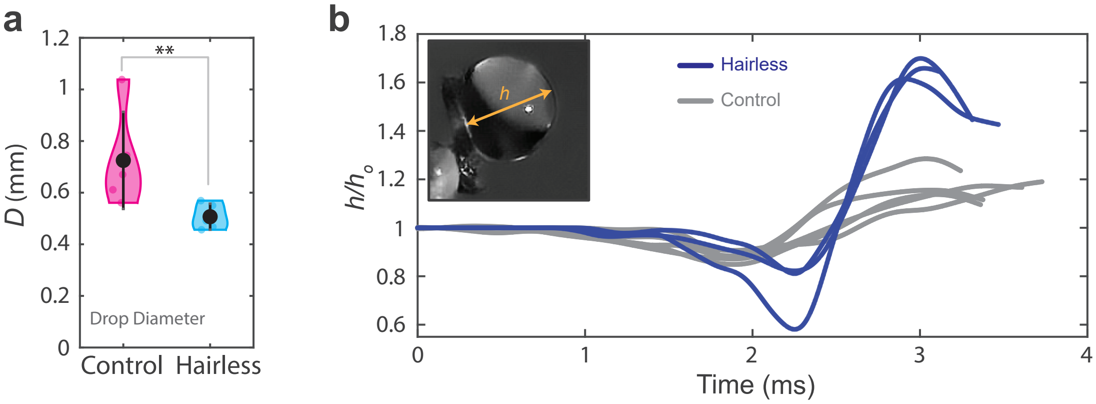

Fig. S4: Droplet size and dynamics in hairless GWSS a. Hairless sharpshooters form droplets having smaller diameters than control sharpshooters. Two-tailed Mann-Whitney test, $**p = 0.016 < 0.02$. $n = 5$ Control; $n = 5$ Hairless, Mean of means ± Standard deviation) **b.** During droplet ejection, droplets experience larger deformation with h/h_o reaching up to $1.6 - 1.8$

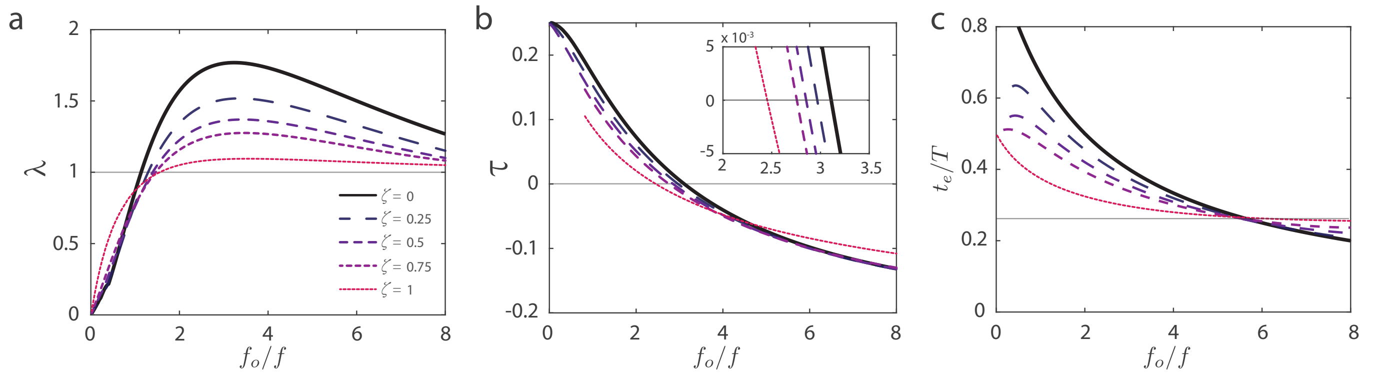

Fig. S5: Effect of damping on ejection kinematics in the two-spring model a. Speed ratio $\lambda = V_d/V_s$ **b.** Compression time and maximum velocity $\tau = (t_c - t_v)/T$ **c.** normalized ejection time t_e/T

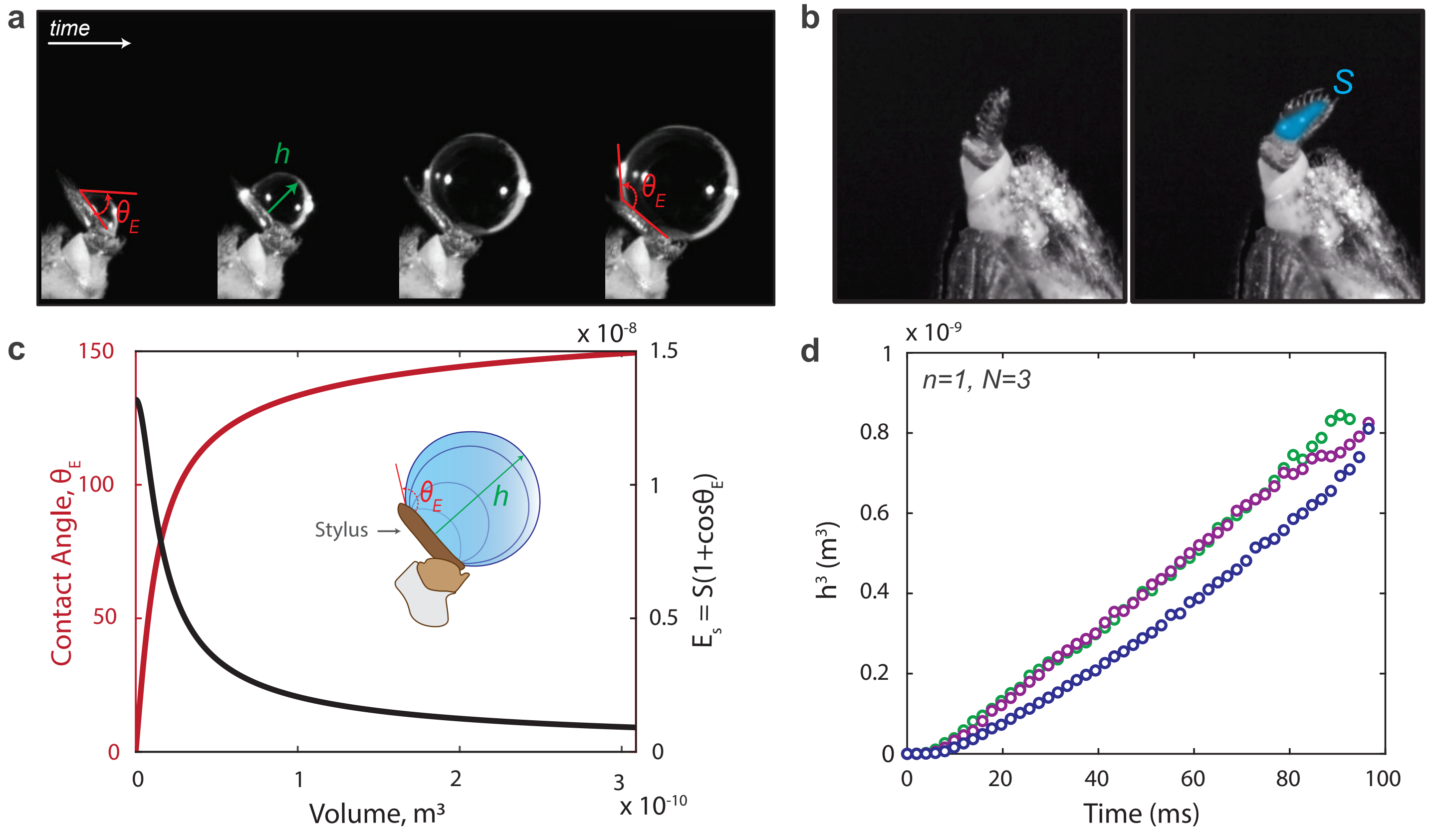

Fig. S6: Droplet growth on parahydrophobic and patterned stylus Droplets are formed on the anal stylus on a constant contact area S. The apparent contact angle θ_a ($\approx \theta_e$) increases as the size of the droplet increase. **b.** Highlighting the hydrophilic area of contact S between the droplet and the stylus (Hairless sharpshooters). **c.** The energy of adhesion decreases as the droplet grows **d.** The droplet growth approximated by $h^3(t)$ is constant, indicating a constant flow rate

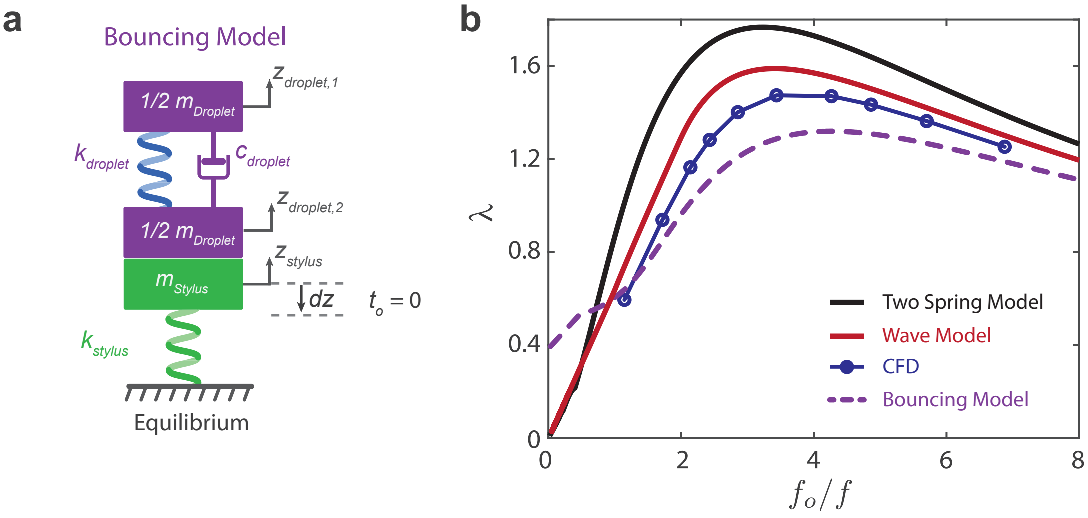

Fig. S7: Comparison between different droplet models for superpropulsion a. Bouncing model adapted from [14] that models the deformation of the droplet using a spring-damper connected between two half-masses (Kelvin-Voight model). The contact force between the lower and upper systems is modeled using a very stiff spring. **b.** Superpropulsion is predicted with different reduced-order models and CFD. The wave model is adapted from [42].

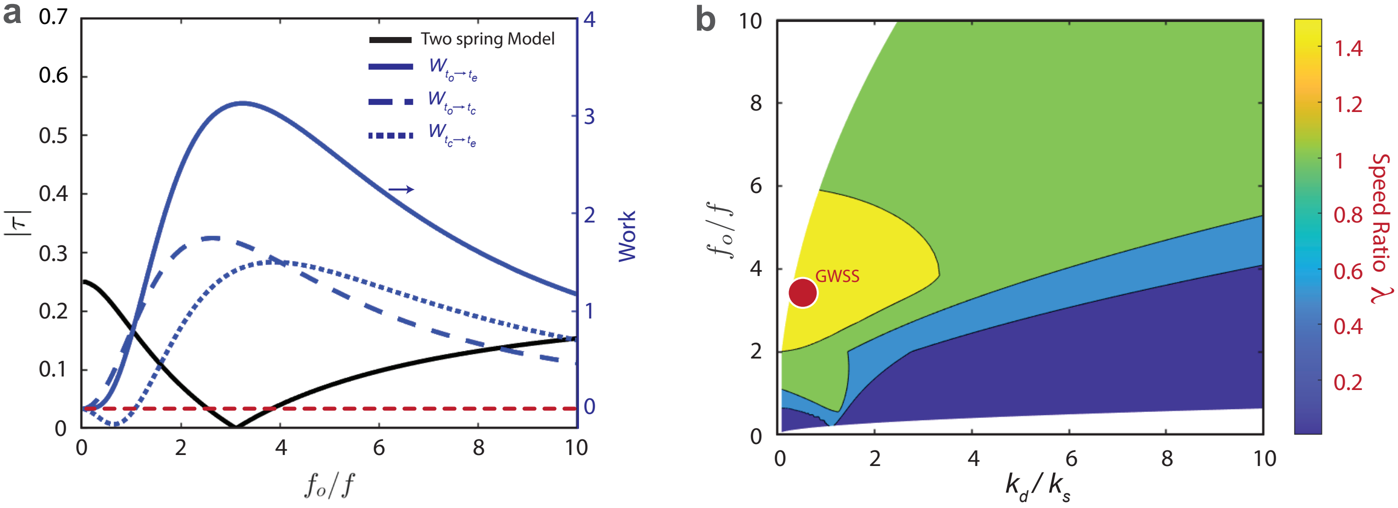

Fig. S8: Energy transfer and coupling limits predicted by the two spring model: a. Energy transfer (work) normalized between t_o, t_c, and t_e: $\bar{W}_{t_o \to te} = \bar{W}_{t_c \to te} + \bar{W}_{t_c \to te}$. The work $\bar{W}_{t_c \to te}$ is negative at low f_o/f **b.** Peak superpropulsion occurs within low coupling between the stiffness ratio k_d/k_s set by surface tension and resilin stiffness.

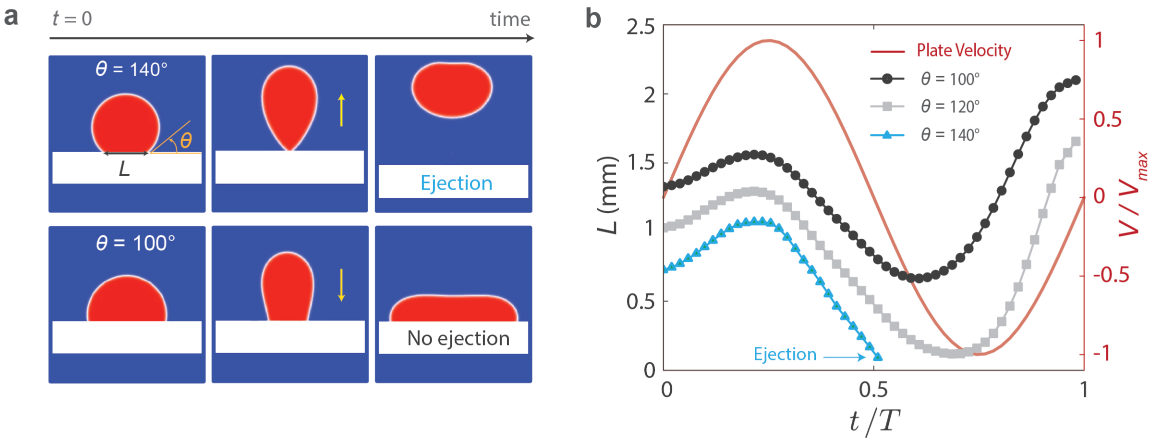

Fig. S9: Dynamics of droplet ejection on a vertically vibrating substrate having different contact angles: a. Detachment of droplets from the surface of a vibrating substrate requires that the contact line L goes to zero while the relative speed between the droplet and the substrate is ≥ 0. **b.** Tracking L over the period of oscillations. At a contact angle of $120°$, the contact line L shrinks to a minimum during droplet extension but does not reach zero (separation).

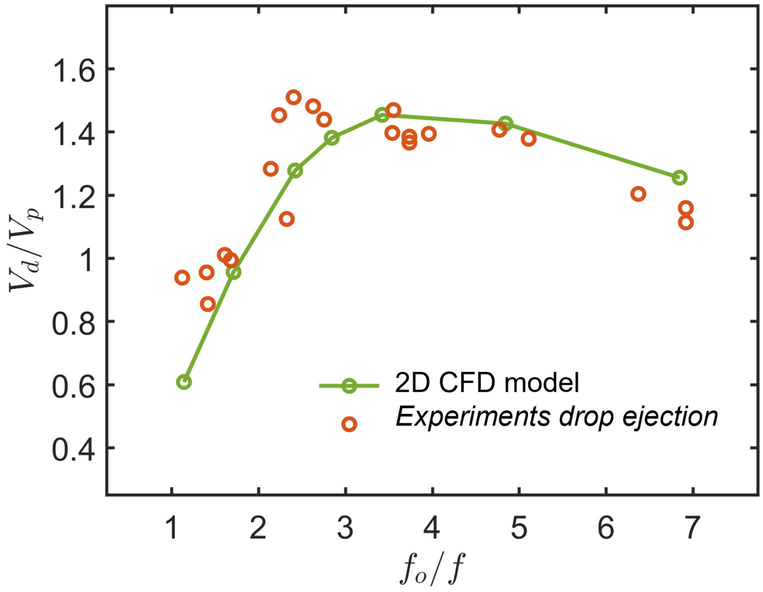

Fig. S10: Matching between the CFD simulation and droplet propulsion from Raufaste *et al.*, PRL 2018 [42].

SUPPLEMENTARY REFERENCES

[1] N. A. Hummel, W. S. Leal, and F. G. Zalom, Potentially hygroreceptive sensilla on the anal stylus of the glassy-winged sharpshooter, homalodisca vitripennis, J Insect Sci **8**, 1 (2008).

[2] S. Kramer, *The morphology and phylogeny of auchenorhynchous Homoptera (Insecta)*, Vol. 20, no. 4 (Urbana,University of Illinois Press,) p. 136, https://www.biodiversitylibrary.org/bibliography/50340.

[3] R. M. Alexander, *Elastic mechanisms in animal movement* (Cambridge University Press, New York, 1988).

[4] J. Michels and S. N. Gorb, Detailed three-dimensional visualization of resilin in the exoskeleton of arthropods using confocal laser scanning microscopy, J Microsc **245**, 1 (2012).

[5] A. B. Gurney, J. P. Kramer, and G. C. Steyskal, Some Techniques for the Preparation, Study, and Storage in Microvials of Insect Genitalia, Annals of the Entomological Society of America **57**, 240 (1964).

[6] A. Mathis, P. Mamidanna, K. M. Cury, T. Abe, V. N. Murthy, M. W. Mathis, and M. Bethge, Deeplabcut: markerless pose estimation of user-defined body parts with deep learning, Nature Neuroscience **21**, 1281 (2018).

[7] N. A. Hummel, W. S. Leal, and F. G. Zalom, Potentially hygroreceptive sensilla on the anal stylus of the glassy-winged sharpshooter, homalodisca vitripennis, J Insect Sci **8**, 1 (2008).

[8] D. Terwagne, F. Ludewig, N. Vandewalle, and S. Dorbolo, The role of the droplet deformations in the bouncing droplet dynamics, Physics of Fluids **25**, 10.1063/1.4832975 (2013).

[9] J. S. Sharp, D. J. Farmer, and J. Kelly, Contact angle dependence of the resonant frequency of sessile water droplets, Langmuir **27**, 9367 (2011), pMID: 21682292.

[10] R. Rakitov, What are brochosomes for? an enigma of leafhoppers (hemiptera, cicadellidae) (2006).

[11] R. Rakitov and S. N. Gorb, Brochosomal coats turn leafhopper (insecta, hemiptera, cicadellidae) integument to superhydrophobic state, Proceedings of the Royal Society B: Biological Sciences **280**, 20122391 (2013).

[12] J. B. Boreyko and C.-H. Chen, Restoring superhydrophobicity of lotus leaves with vibration-induced dewetting, Phys. Rev. Lett. **103**, 174502 (2009).

[13] K. Okumura, F. Chevy, D. Richard, D. Quéré, and C. Clanet, Water spring: A model for bouncing drops, Europhysics Letters (EPL) **62**, 237 (2003).

[14] W. Wang, C. Ji, F. Lin, J. Zou, and S. Dorbolo, Water drops bouncing off vertically vibrating textured surfaces, Journal of Fluid Mechanics **876**, 1041–1051 (2019).

[15] M. Hubert, D. Robert, H. Caps, S. Dorbolo, and N. Vandewalle, Resonant and antiresonant bouncing droplets, Phys Rev E Stat Nonlin Soft Matter Phys **91**, 023017 (2015).

[16] M. R. Weiss, Defecation behavior and ecology of insects, Annu Rev Entomol **51**, 635 (2006).

[17] C. Nalepa, D. Bignell, and C. Bandi, Detritivory, coprophagy, and the evolution of digestive mutualisms in dictyoptera, Insectes Sociaux **48**, 194–201 (2001).

[18] T. Eisner and M. Eisner, Defensive use of a fecal thatch by a beetle larva (hemisphaerota cyanea), Proceedings of the National Academy of Sciences **97**, 2632–2636 (2000).

[19] R. Fettköther, G. V. Reddy, U. Noldt, and K. Dettner, Effect of host and larval frass volatiles on behavioural response of the old house borer, hylotrupes bajulus (l.) (coleoptera: Cerambycidae), in a wind tunnel bioassay, Chemoecology **10**, 1–10 (2000).

[20] P. J. Yang, J. Pham, J. Choo, and D. L. Hu, Duration of urination does not change with body size, Proceedings of the National Academy of Sciences **111**, 11932–11937 (2014).

[21] D. Horsfield, Evidence for xylem feeding by philaneus spumarius (l.) (homoptera: Cercopidae), Entomologia Experimentalis et Applicata **24**, 95 (1978).

[22] T. E. Mittler, Water Tensions in Plants—an Entomological Approach1, Annals of the Entomological Society of America **60**, 1074 (1967).

[23] P. C. Andersen, B. V. Brodbeck, and R. F. Mizell III, Metabolism of amino acids, organic acids and sugars extracted from the xylem fluid of four host plants by adult homalodisca coagulata, Entomologia Experimentalis et Applicata **50**, 149 (1989).

[24] V. Novotny and M. R. Wilson, Why are there no small species among xylem-sucking insects?, Evolutionary Ecology **11**, 419 (1997).

[25] F. Zhang, C. Zhang, W. Dai, and Y. Zhang, Morphology and histology of the digestive system of the vector leafhopper *Psammotettix* striatus (*L.*) (hemiptera: Cicadellidae), Micron **43**, 725 (2012).

[26] V. Storch, B. Bluhm, and W. Arntz, Microscopic anatomy and ultrastructure of the digestive system of three Antarctic shrimps (Crustacea: Decapoda: Caridea), Polar Biology **24**, 604 (2001).

[27] P. C. Andersen, B. V. Brodbeck, and R. F. Mizell, Feeding by the leafhopper, homalodisca coagulata, in relation to xylem fluid chemistry and tension, Journal of Insect Physiology **38**, 611 (1992).

[28] A. H. Purcell, A. H. Finlay, and D. L. McLean, Pierce's disease bacterium: Mechanism of transmission by leafhopper vectors, Science **206**, 839–841 (1979).

[29] L. C. Berlin and E. T. Hibbs, Digestive system morphology and salivary enzymes of the potato leafhopper, empoasca fabae (harris) leafhopper, empoasca fabae (harris), Proceedings of the Iowa Academy of Science **70**, 527–540 (1963).

[30] P.-G. de Gennes, F. Brochard-Wyart, and D. Quéré, *Capillarity and wetting phenomena: drops, bubbles, pearls, waves* (Springer, 2004).

[31] T. Hasegawa, M. Suganuma, and H. Watanabe, Anomaly of excess pressure drops of the flow through very small orifices, Physics of Fluids **9**, 1–3 (1997).

[32] N. M. Smith, H. Ebrahimi, R. Ghosh, and A. K. Dickerson, High-speed microjets issue from bursting oil gland reservoirs of citrus fruit, Proceedings of the National Academy of Sciences **115**, E5887 (2018).

[33] T. Hasegawa, A. Ushida, and T. Narumi, Anomalous reduction in pressure drops of the water flow through micro-orifices in high velocity ranges, AIP Conference Proceedings **1027**, 991 (2008).

[34] W. Kim and J. W. M. Bush, Natural drinking strategies, Journal of Fluid Mechanics **705**, 7–25 (2012).

[35] K. I. Beckett, A. B. Robertson, and P. G. Matthews, Studies on gas exchange in the meadow spittlebug, philaenus spumarius: the metabolic cost of feeding on, and living in, xylem sap, Journal of Experimental Biology **222**, 10.1242/jeb.191973 (2019).

[36] E. A. Bergman, E. L. Green, and P. G. D. Matthews, The cibarial pump of the xylem-feeding froghopper (*Philaenus spumarius*) produces negative pressures exceeding 1 mpa, Proceedings of the Royal Society B: Biological Sciences **288**, 20210731 (2021).

[37] W. van Hoeve, S. Gekle, J. H. Snoeijer, M. Versluis, M. P. Brenner, and D. Lohse, Breakup of diminutive rayleigh jets, Physics of Fluids **22**, 122003 (2010).

[38] C. Clanet and J. C. Lasheras, Transition from dripping to jetting, Journal of Fluid Mechanics **383**, 307–326 (1999).

[39] L. Yafetto, L. Carroll, Y. Cui, D. J. Davis, M. W. F. Fischer, A. C. Henterly, J. D. Kessler, H. A. Kilroy, J. B. Shidler, J. L. Stolze-Rybczynski, Z. Sugawara, and N. P. Money, The fastest flights in nature: High-speed spore discharge mechanisms among fungi, PLOS ONE **3**, 1 (2008).

[40] A. Pringle, S. N. Patek, M. Fischer, J. Stolze, and N. P. Money, The captured launch of a ballistospore, Mycologia **97**, 866 (2005), pMID: 16457355.

[41] A. Vailati, L. Zinnato, and R. Cerbino, How archer fish achieve a powerful impact: Hydrodynamic instability of a pulsed jet in toxotes jaculatrix, PLOS ONE **7**, 1 (2012).

[42] C. Raufaste, G. R. Chagas, T. Darmanin, C. Claudet, F. Guittard, and F. Celestini, Superpropulsion of droplets and soft elastic solids, Phys. Rev. Lett. **119**, 108001 (2017).
