# Supplementary material for: Droplet superpropulsion in an energetically constrained insect
Source: Nat Commun. 2023 Feb 28;14:860. doi: 10.1038/s41467-023-36376-5 (PMC9975225; doi:10.1038/s41467-023-36376-5)
Supplement: Supplementary file 3 — Description of Additional Supplementary Files [file 41467_2023_36376_MOESM3_ESM.pdf]

File name: Supplementary Video I

Description: a- Real-time video of a glassy-winged sharpshooter insect ejecting droplets, **b-** High-speed video (4484 fps) of blue-green sharpshooter insect highlighting the different phases of droplet ejection, **c-** Accumulation of 'pee' puddle at large distances due to continuous droplet ejection, d Parabolic trajectory, and repeatability of droplet ejections, **e**- Manual manipulation, and elasticity of anal stylus, **f**- Manual manipulation and elasticity of anal stylus, **g-** Dynamics of droplets in control vs. hairless sharpshooters. Droplet deformation is larger during droplet ejection in sharpshooters.

File name: Supplementary Video II

Description: Drop-on-plate and computational fluid dynamic (CFD) simulations **a**- Tracking the centroid of the droplet and plate during drop-on-plate experiments, **b**- CFD simulations of 1 mm droplets on a vibrating plate (constant contact angle of $\sim 180^{\circ}$, Variable frequencies), **c**- CFD simulations of 1 mm droplets on a vibrating plate (Constant frequency of 100 Hz, variable contact angle).

File name: Supplementary Video III

Description: a- MicroCT imaging of sharpshooter insect a 3D reconstruction of the side view of sharpshooter hindgut, **b**- Side view 2D cross-section of GWSS hindgut, **c-** Top view 2D cross-section of GWSS hindgut.
