# Supplementary material for: Droplet superpropulsion in an energetically constrained insect
Source: Nat Commun. 2023 Feb 28;14:860. doi: 10.1038/s41467-023-36376-5 (PMC9975225; doi:10.1038/s41467-023-36376-5)
Supplement: Supplementary file 7 — Reporting Summary [file 41467_2023_36376_MOESM7_ESM.pdf]

nature portfolio

Corresponding author(s): Saad Bhamla

Last updated by author(s): Jan 15, 2023

Reporting Summary

Nature Portfolio wishes to improve the reproducibility of the work that we publish. This form provides structure for consistency and transparency in reporting. For further information on Nature Portfolio policies, see our Editorial Policies and the Editorial Policy Checklist.

Statistics

For all statistical analyses, confirm that the following items are present in the figure legend, table legend, main text, or Methods section.

n/a	Confirmed	
☐	☒	The exact sample size (*n*) for each experimental group/condition, given as a discrete number and unit of measurement
☐	☒	A statement on whether measurements were taken from distinct samples or whether the same sample was measured repeatedly
☐	☒	The statistical test(s) used AND whether they are one- or two-sided<br>*Only common tests should be described solely by name; describe more complex techniques in the Methods section.*
☐	☒	A description of all covariates tested
☐	☒	A description of any assumptions or corrections, such as tests of normality and adjustment for multiple comparisons
☐	☒	A full description of the statistical parameters including central tendency (e.g. means) or other basic estimates (e.g. regression coefficient) AND variation (e.g. standard deviation) or associated estimates of uncertainty (e.g. confidence intervals)
☐	☒	For null hypothesis testing, the test statistic (e.g. F, t, r) with confidence intervals, effect sizes, degrees of freedom and P value noted<br>*Give P values as exact values whenever suitable.*
☒	☐	For Bayesian analysis, information on the choice of priors and Markov chain Monte Carlo settings
☒	☐	For hierarchical and complex designs, identification of the appropriate level for tests and full reporting of outcomes
☒	☐	Estimates of effect sizes (e.g. Cohen's *d*, Pearson's *r*), indicating how they were calculated

Our web collection on statistics for biologists contains articles on many of the points above.

Software and code

Policy information about availability of computer code

Data collection	A high-speed camera (Chronos 1.4) was used in this study to image the organisms. COMSOL Multiphysics 5.6 was used to perform the droplet-on-plate computational experiments.
Data analysis	Tracking was done with ImageJ (1.53v) Data analysis and plotting were done using MATLAB 2022.<br>The source data and MATLAB codes used to generate the superpropulsion plots may be found here: https://github.com/bhamla-lab/Sharpshooter_Nat_Comm_2023. The description of all the other data analysis techniques is provided in the main manuscript and supplementary documents.

For manuscripts utilizing custom algorithms or software that are central to the research but not yet described in published literature, software must be made available to editors and reviewers. We strongly encourage code deposition in a community repository (e.g. GitHub). See the Nature Portfolio guidelines for submitting code & software for further information.

Data

Policy information about availability of data

All manuscripts must include a data availability statement. This statement should provide the following information, where applicable:

- Accession codes, unique identifiers, or web links for publicly available datasets
- A description of any restrictions on data availability
- For clinical datasets or third party data, please ensure that the statement adheres to our policy

There are no restrictions that exist on data availability. The datasets for all the presented plots and simulations are present in the main manuscript and github: https://github.com/bhamla-lab/Sharpshooter_Nat_Comm_2023

Human research participants

Policy information about studies involving human research participants and Sex and Gender in Research.

Reporting on sex and gender	N/A
Population characteristics	N/A
Recruitment	N/A
Ethics oversight	N/A

Note that full information on the approval of the study protocol must also be provided in the manuscript.

Field-specific reporting

Please select the one below that is the best fit for your research. If you are not sure, read the appropriate sections before making your selection.

☒ Life sciences ☐ Behavioural & social sciences ☐ Ecological, evolutionary & environmental sciences

For a reference copy of the document with all sections, see nature.com/documents/nr-reporting-summary-flat.pdf

Life sciences study design

All studies must disclose on these points even when the disclosure is negative.

Sample size	The sample size of the number of insects and the number of fluidic shooting events were chosen based on the availability of the GWSS and BGSS samples at USDA, as explained within the manuscript and supplementary information. Data were collected while ensuring that the full movement of the stylus and droplet remains in a 2D plane. Given the difficulties of collecting data in the wild, the data for red-banded sharpshooters were limited to one sample with N=3 shooting events, as explained in the manuscript. Fig 1e-f: The kinematics data were shown from a single individual to highlight the repeatability of the stylus moving per individual. Fig 1g and the rest of the figures: The speed ratio is shown for all n=5 GWSS individuals with N=22 shooting events. Our observation of these individuals and shooting events revealed consistent results. Fig 3: The hairless sharpshooters n=5 with N=10 shooting events led to the same conclusion that hairless sharpshooters were in the subpropulsion regime. Fig 4: All GWSS data were considered during the pressure calculations.
Data exclusions	Data of fluidic shooting events where the stylus and/or droplet went out of the shooting frame were excluded from calculations.
Replication	Three of the authors collected the data presented in this paper on three different occasions. The data were analyzed three times by three different individuals to reduce potential biases. All attempts at replications were successful and agreed with the conclusions of the paper.
Randomization	Individual insects were chosen randomly chosen from their enclosure at for imaging To ensure randomness, different individuals were imaged in different enclosures. For the hairless sharpshooter experiments, at least 5 individual adult insects were picked randomly (regardless of age and size) and placed in a separate enclosure.
Blinding	Blinding was not relevant for our study since no a-prior grouping was done on insects used in the experiments.

Reporting for specific materials, systems and methods

We require information from authors about some types of materials, experimental systems and methods used in many studies. Here, indicate whether each material, system or method listed is relevant to your study. If you are not sure if a list item applies to your research, read the appropriate section before selecting a response.

Materials & experimental systems

n/a	Involved in the study
☒	☐ Antibodies
☒	☐ Eukaryotic cell lines
☒	☐ Palaeontology and archaeology
☐	☒ Animals and other organisms
☒	☐ Clinical data
☒	☐ Dual use research of concern

Methods

n/a	Involved in the study
☒	☐ ChIP-seq
☒	☐ Flow cytometry
☒	☐ MRI-based neuroimaging

Animals and other research organisms

Policy information about studies involving animals; ARRIVE guidelines recommended for reporting animal research, and Sex and Gender in Research

Laboratory animals	This study involves glassy-winged sharpshooter insects (GWSS) and blue-green sharpshooter insects (BGSS) which were imaged at the United States Department of agriculture (USDA) facilities at Parlier, California, USA.
Wild animals	This study involves red-banded sharpshooter insects that were observed and imaged in Atlanta, GA, USA.
Reporting on sex	These findings are not specific to one sex.
Field-collected samples	This study involves capturing red-banded sharpshooter insects and storing them in a 70% ethanol solution.
Ethics oversight	This study did not require any ethics approval or guidance.

Note that full information on the approval of the study protocol must also be provided in the manuscript.
